# Neonatal Immunization with a Single IL-4/Antigen Dose Induces Increased Antibody Responses after Challenge Infection with Equine Herpesvirus Type 1 (EHV-1) at Weanling Age

**DOI:** 10.1371/journal.pone.0169072

**Published:** 2017-01-03

**Authors:** Bettina Wagner, Gillian Perkins, Susanna Babasyan, Heather Freer, Alison Keggan, Laura B. Goodman, Amy Glaser, Sigurbjorg Torsteinsdóttir, Vilhjálmur Svansson, Sigríður Björnsdóttir

**Affiliations:** 1 Department of Population Medicine and Diagnostic Sciences, College of Veterinary Medicine, Cornell University, Ithaca, NY, United States of America; 2 Department of Clinical Sciences, College of Veterinary Medicine, Cornell University, Ithaca, NY, United States of America; 3 Institute for Experimental Pathology, Keldur, University of Iceland, Reykjavik, Iceland; 4 Icelandic Food and Veterinary Authority, MAST, Office of Animal Health and Welfare, Selfoss, Iceland; Midwestern University, UNITED STATES

## Abstract

Neonatal foals respond poorly to conventional vaccines. These vaccines typically target T-helper (Th) cell dependent B-cell activation. However, Th2-cell immunity is impaired in foals during the first three months of life. In contrast, neonatal basophils are potent interleukin-4 (IL-4) producers. The purpose of this study was to develop a novel vaccine triggering the natural capacity of neonatal basophils to secrete IL-4 and to evaluate if vaccination resulted in B-cell activation and antibody production against EHV-1 glycoprotein C (gC). Neonatal vaccination was performed by oral biotinylated IgE (IgE-bio) treatment at birth followed by intramuscular injection of a single dose of streptavidin-conjugated gC/IL-4 fusion protein (Sav-gC/IL-4) for crosslinking of receptor-bound IgE-bio (group 1). Neonates in group 2 received the intramuscular Sav-gC/IL-4 vaccine only. Group 3 remained non-vaccinated at birth. After vaccination, gC antibody production was not detectable. The ability of the vaccine to induce protection was evaluated by an EHV-1 challenge infection after weaning at 7 months of age. Groups 1 and 2 responded to EHV-1 infection with an earlier onset and overall significantly increased anti-gC serum antibody responses compared to control group 3. In addition, group 1 weanlings had a decreased initial fever peak after infection indicating partial protection from EHV-1 infection. This suggested that the neonatal vaccination induced a memory B-cell response at birth that was recalled at weanling age after EHV-1 challenge. In conclusion, early stimulation of neonatal immunity via the innate arm of the immune system can induce partial protection and increased antibody responses against EHV-1.

## Introduction

Equine herpesvirus type-1 (EHV-1) is member of the *Varicellovirus* genus in the *Alphaherpesvirinae* subfamily that is highly prevalent in most equine populations [1–3]. EHV-1 has a substantial impact on equine health and equine industries worldwide through respiratory disease, abortion, and encephalomyelopathy [3,4]. Most horses are first infected early in life and are believed to remain latently infected for life. Latently infected, lactating mares act as source of infection for their foals after birth, which in turn infect other foals and weanlings [1,2,5]. The virus is spread with respiratory secretions via direct nose-to-nose contact or contact with fomites. EHV-1 first infects the respiratory epithelium and quickly enters the retropharyngeal lymphoid tissues. From here the virus spreads systemically via a cell-associated viremia, and latency is established in the trigeminal ganglion and possibly some long-lived immune cells [6,7]. EHV-1 is reactivated from the latent state and is shed again during times of stress, and all clinical manifestations may be seen during recrudescence [3].

Currently, a number of vaccines against EHV-1, including inactivated and modified-live virus vaccines, are available in the US and Europe. Through the use of these vaccines the occurrence of abortion storms has generally decreased. However, EHV-1 outbreaks continue to occur despite widespread vaccination [3,6,8]. Because very young foals react weakly to available vaccines, first vaccination is generally recommended between 4–6 months of age [9]. For pathogens that are frequently transmitted early in life such as EHV-1, this vaccination regime does not appear to be effective in preventing the initial EHV-1 infection of foals and consequently the establishment of latent infection early in life.

All currently available EHV-1 vaccines are optimized to induce immunity in adult horses. This is achieved by stimulating B-cells and antibody responses via Th-cell mediated pathways. During their interaction with B-cells antigen-specific Th2-cells produce the cytokine interleukin-4 (IL-4), formerly called B-cell stimulatory factor 1 [10]. IL-4 then initiates B-cell differentiation and immunoglobulin class switching which results in formation of antigen-specific memory B-cells and IgG secreting plasma cells.

Different research groups, including ours, have shown that T-cell responses and cytokine production from T-cells are reduced and delayed in foals for at least three months after birth [11–13]. We have previously shown that Th2-responses are almost undetectable in neonates. IL-4 production by Th2-cells increased slowly with age and did not reach adult levels by three months of age [13]. The findings offered an explanation for the weak immune response of foals to currently available vaccines because induction of a classical Th2-response is a key mechanism of most vaccines for initiating robust antibody titers.

Nevertheless, IgG antibody production occurs in foals before or shortly after birth for most IgG isotypes [14]. Foal IgG1 is first produced in utero, can be detected after birth before colostrum uptake, exceeds maternal IgG1 by 5 weeks of age, and is up to 3-fold higher in serum of 2–3 months old foals compared to adult horse serum [15,16]. While maternal antibodies decline, endogenous IgG3/5, and IgA synthesis were detectable within the first five to eight weeks of life and reached adult serum levels by 3 months of age [15,16]. This suggests that IgG3/5 and IgA production started within the first 2–3 weeks of life [14]. Only IgG4 antibodies showed a delay of endogenous production in foals [16], and this may functionally be compensated by the increased IgG1 concentrations in young foals. This suggests that IgG1 and IgG3/5 production start in foals before Th2-immunity matures. The disconnection of IgG production and Th2-immunity early in life suggests that neonatal B-cell stimulation and antibody production are initiated by Th2-independent, alternative pathways.

We have previously shown that IL-4 is produced in neonatal foals by basophils [17]. Neonatal foal basophils become equipped with Fc-receptor bound IgE within the first few hours after birth. These IgE antibodies originate from the dam, are transferred to the neonate in the colostrum, and can be found on basophils in the neonatal circulation for the first 2–3 months of life [18]. Crosslinking of receptor-bound IgE leads to profound IL-4 production in, and secretion from, the neonatal basophils. IL-4 production by neonatal basophils peaked at day 5 of age [16] and can be induced during the first three months of life by IgE receptor crosslinking or non-specific stimulation of basophils, while IL-4 production by Th2-cells is lacking [13]. In adult people, basophils are potent IL-4 producers [19]. Various groups have shown in adult humans or rodent models that basophils have important immune regulatory functions for the induction of innate and adaptive immunity including the development of Th2-cells and the regulation of antibody production [20–23].

Based on these findings, we hypothesized that neonatal basophil-derived IL-4 can directly activate B-cell differentiation and provide a Th2-independent stimulation pathway for IgG production in neonatal and young foals. Here, we evaluated a novel neonatal vaccine for immunity and protection against EHV-1 infection by targeting IL-4 production in basophils to induce B-cell activation after birth. The neonatal EHV-1 vaccine was composed of EHV-1 glycoprotein C (gC) antigen. EHV-1 gC is highly immunogenic in mouse models and the natural hosts [24–26], is involved in cell entry [27,28], was targeted for vaccine development in rodents [29], and is able to interfere with the activation of the complement cascade by binding to the complement component C3 [30]. EHV-1 gC-specific basophils and B-cell activation was accomplished by oral treatment with biotinylated IgE (IgE-bio), which was then cross-linked on the basophil surface with EHV-1 gC antigen by using a streptavidin-conjugated gC/IL-4 fusion protein (Sav-gC/IL-4). Neonatal foals received this vaccination at birth. An experimental EHV-1 challenge was performed at weanling age, a time of stress and thus increased risk for EHV-1 transmission and disease, to test if the neonatal vaccination provided enhanced immunity and thus protection against EHV-1 infection.

## Materials and Methods

### EHV-1 naïve foals

Fifteen neonatal foals from an EHV-1-free herd of Icelandic horses at Cornell University were enrolled in this study. EHV-1 has not been reported in Iceland [31]. General and targeted surveillance by PCR testing from abortions, respiratory or neurological cases in Iceland have been negative for EHV-1 since monitoring began in 1990 (personal communication Dr. Vilhjálmur Svansson, University of Iceland, Keldur, Reykjavík). In contrast, EHV-4 occurs in Iceland. The neonatal foals were offspring of 15 pregnant mares that were purchased from private owners, bred in Iceland, and imported from Iceland to the US while 6–7 months pregnant in February 2012. Import quarantine for the mares was performed at an isolated facility at Cornell University under USDA/APHIS supervision. All horses were kept at this facility and had no contact to other horses in the US prior to and for the duration of this study. The facility had restricted access for people to avoid infection with common US pathogens and to maintain the EHV-1-free status of the Icelandic herd. The mares were vaccinated against rabies and tetanus after the import quarantine and then annually. They were and dewormed on a regular basis. The mares were not vaccinated against EHV-1 or EHV-4 before foaling. The 15 foals were born at the isolated facility within 3 weeks in June 2012 without complications. All foals suckled colostrum ad libatum. Due to the specific EHV-1-free status of the mares, the colostrum did not contain EHV-1-specific maternal antibodies at birth. At birth, foals were randomly assigned to three groups. All foals were experimentally challenged with EHV-1 at weanling age as outlined below. After birth and before experimental EHV-1 infection, all foals were kept on pasture with their mares. Grass hay was fed to all horses ad libatum. All foals were clinically healthy from birth to weanling age. They were first vaccinated against tetanus and rabies and dewormed with ivermectin at 6 months of age but were not vaccinated or treated otherwise. The neonatal vaccination, the experimental EHV-1 challenge and all sample collections for this study were carried out in accordance with the recommendation in the Guide for the Care and Use of Laboratory Animals of the National Institute of Health. The protocol was approved by the Institutional Animal Care and Use Committee at Cornell University (protocol #2011–0011). Since the study was performed in horses the work also followed the Guide for Care and Use of Animals in Agricultural Research and Teaching. All efforts were made to minimize suffering of the animals for example by administering tranquilizers for nervous or excited horses before sampling. After the end of this experimental study, all horses were kept at the facility at Cornell University as research horses.

### Neonatal EHV-1 vaccination

Fifteen neonatal foals were divided into three groups of five foals each. Group 1 (3 fillies, 2 colts) received IgE-bio at birth and before colostrum intake. The purification and biotinylation of equine IgE [32,33] was performed as described in S1 File. A total of 1 mg IgE-bio was given via nasogastric intubation in a total of 20–30 ml 0.9% saline solution. Group 2 (2 fillies, 3 colts) did not receive IgE-bio at birth. On day 2 of life, group 1 and group 2 foals received an intramuscular (IM) injection of 0.5 mg Sav-gC/IL-4 into the left semitendonosus muscle. The expression and purification of gC/IL-4 was performed as previously described [26,34]. Sav coupling was described in detail in S1 File. Group 3 foals (1 filly, 4 colts) received neither IgE-bio nor Sav-gC/IL-4 antigen.

### Blood sample collection after neonatal vaccination

Blood samples were collected by jugular venipuncture with a vacutainer collection system and a 20-gauge needle. Blood tubes without coagulant or with heparin were used for serum collection or peripheral blood mononuclear cell (PBMC) isolation, respectively. Serum samples for antibody measurements were collected from all foals immediately after birth before IgE was administrated (group 1) and before colostrum intake (all groups), on days 2 (before EHV-1 gC/IL-4 was given), 5, 12, 25 and 60 and 90 of age. Blood in tubes without coagulant was allowed to clot and after centrifugation (3,000xg, RT, 15 min). Serum was collected and frozen at –20°C for later measurements of EHV-1-specific antibodies in serum. Heparinized blood samples were obtained from neonatal foals on days 2 and 5 after birth for PBMC isolation and basophil stimulation (S1 File).

### EHV-1 Multiplex assay for EHV-1-specific total antibody and isotype detection

An EHV-1 Multiplex assay was used for measuring total EHV-1 gC and gD-specific antibodies in serum samples after neonatal vaccination. The EHV-1 Multiplex assay was also used to measure EHV-1 specific isotype responses in serum samples after experimental EHV-1 infection at weanling age. The EHV-1 Multiplex assay for gC and gD antigens was described previously in detail [26]. The assay correlates highly to EHV-1 SN-testing but has a much wider linear quantification range and allows for Ig isotype analysis.

### Experimental EHV-1 challenge infection at weanling age

At 7 months of age, the foals were weaned and moved to a separate pasture. Two days later (d-2), foals were moved into the isolation barn with individual box stalls and allowed to acclimate for two days. The stalls did not allow the horses to have direct nose-to-nose contact. However, the barn had a center hallway with the same airspace and horses were handled in the facility as one group. Biosecurity precautions were taken by people upon entering and exiting the facility. No other specific care was taken to prevent spread of virus from horse-to-horse by animal handlers. Baseline serum and nasal secretion samples were taken the day before EHV-1 infection (d-1). Baseline physical examination measurements were taken a day prior to and immediately before EHV-1 infection on d0. Then, all horses were infected intranasally with 1 x 10^7^ plaque-forming units (PFU) of EHV-1 strain NY03 in 7 ml of saline with a mucosal atomizer device (Wolfe Tory Medical, Salt Lake City, UT). The EHV-1 strain NY03 does not possess the D752-encoding single nucleotide polymorphism and is typed as a “non-neurogenic” strain [35].

### Clinical evaluation

Temperatures were taken in the morning and evening until day 7 post infection (pi) and then once a day before samples were obtained in the morning. A fever was defined as a rectal temperature of >101.5°F (> 38.6°C). Clinical scoring was performed daily until day 17 pi by an equine clinician who was unaware of the treatment at birth or the group assignments. Scoring was performed according to a clinical scoring system described by Furr and coworkers [36] and included individual scores for nasal discharge, eye discharge, mandibular lymph nodes and a neurologic score resulting in a daily total clinical score for each horse. Neurologic scoring was performed by walking the horses in the aisle way of the isolation barn to assess for ataxia.

### Blood and nasal secretion samples after experimental EHV-1 infection

Blood samples were collected on days -1, 1 to 10, 12, 14, 19, 26, 40, 61, 122, 150, 172 and 220 pi. Serum samples were used for EHV-1 specific antibody multiplex analysis (until day 220 pi) and for cytokine detection (until day 19 pi). PBMC were isolated from heparinized blood. PBMC aliquots were used immediately for cellular stimulation assays to detect cytokines in supernatants (until day 150 pi) and EHV-1-specific T-cells (until day 220 pi), as described below. Snap frozen aliquots of 5 x 10^6^ PBMC obtained from day -1 until day 14 pi were used for EHV-1 PCR to determine viremia.

Nasal swabs were obtained on days -1, 1 to 10, 12, and 14 pi using two sterile, polyester tipped swabs (Puritan Medical Products Company, LLC, Guilford, ME) placed in the nostril contacting the nasal mucosa for up to 2–3 seconds. The swabs were placed directly into polystyrene tubes containing 1 ml of PBS. Samples were subsequently transported to the laboratory where they were centrifuged at 200xg for 10 min at 4°C and stored in two aliquots immediately afterwards at -80°C. One aliquot containing the pellet plus 200 μl of the solution above the pellet was used for EHV-1 PCR.

### EHV-1 PCR

DNA was isolated from nasal swab samples to determine viral genome copy numbers in nasal secretions by quantitative PCR targeting the gB gene [37] using purified amplicon standards and 4 μl of template (from a 90 μl elution). Aliquots of 5 x 10^6^ snap frozen PBMC pellets were extracted and tested using the same assay with a positive cutoff imposed at the analytic limit of 100% detection. Cycle threshold (Ct) values were calculated based on the automated algorithm on the Applied Biosystems 7500 platform. The quantitative PCR was performed at the Animal Health Diagnostic Center at Cornell University.

### Cytokine and interferon detection by multiplex analysis

Cytokines and interferons (all called cytokines from here on) were determined in cell culture supernatants from equine peripheral blood mononuclear cells (PBMC) using an fluorescent beads-based equine cytokine multiplex assays for IFN-α, IL-4, IL-10, IL-17 and IFN-γ as previously described [38]. The Equine Cytokine Multiplex assay is available through the Animal Health Diagnostic Center at Cornell University. IL-4, IL-10 and IFN-α were expressed in pg/ml and IL-17 and IFN-γ were reported as U/ml.

### *In vitro* re-stimulation of PBMC with EHV-1

EHV-1 re-stimulation of PBMC was performed using the EHV-1 strain RacL11 at a MOI of 1 as previously described [39,40]. The PMA/ionomycin stimulation [39,40] was used as a positive and viability control of the PBMC, and high cytokine values were measured after PMA/ionomycin stimulation for all samples and cytokines described here.

### Flow cytometric analysis of cytokine producing EHV-1 re-stimulated T-cells

For flow cytometric analysis, cells were harvested after 48 hours of stimulation, fixed, permeabilized, and aliquots were triple-stained for (1) cell surface CD4 and CD8, and intracellular IFN-γ expression, or (2) intracellular IL-4, IL-10 and IL-17A expression using directly labeled antibodies and isotype-matched staining controls as previously described [13,40,41]. The cells were analyzed in a FACS Canto II flow cytometer (BD Biosciences, San Diego, CA).

### Statistical analysis

D’Agostino & Pearson normality tests indicated that values on most days were not normally distributed. All post EHV-1 challenge parameters were compared by repeated-measures ANOVAs with Bonferroni post tests between all three neonatal vaccination groups. The intracellular cytokines production by T-cells did not show any significant differences between the three groups and changes were thus considered as not influenced by the neonatal vaccination. Intracellular cytokines production by T-cells was analyzed for all 15 weanlings to describe the changes in their cellular immunity to EHV-1 infection over time by nonparametric Friedman tests for repeated measurements followed by Dunn’s tests. Each of the post infection time points was compared to the pre-infection value. P-values of <0.05 were considered significant. The statistical analysis was performed and the graphs were created using GraphPad Prism 6 for Mac OS X, version 6f.

## Results

### Immune response after neonatal EHV-1 vaccination

The binding of IgE-bio to equine cells, the IL-4 induction after IgE-bio crosslinking, and the Sav-gC/IL-4 conjugation and were first evaluated *in vitro* (S2 File). IgE-bio bound to high-affinity IgE receptors on equine basophils and crosslinking induced IL-4 secretion *in vitro* (S1 Fig). After neonatal vaccination, EHV-1 gC and gD-specific antibody values were measured in sera of all foals immediately after birth, before and after neonatal EHV-1 vaccination until foals were 3 months of age. Differences in gC or gD-specific antibodies were not observed between the three groups (S2 File). In addition, IgE^+^ cells were stimulated *ex vivo* to test if the vaccination resulted in detectable amounts of IL-4 secreting basophils in the circulation of the neonates. While anti-IgE stimulated IL-4 production by basophils, as expected, Sav-gC/IL-4 stimulation did not result in detectable percentages of IgE^+^/IL-4^+^ cells (S2 Fig).

### Clinical signs, nasal EHV-1 shedding and viremia after EHV-1 infection at weanling age

**Fig 1:**
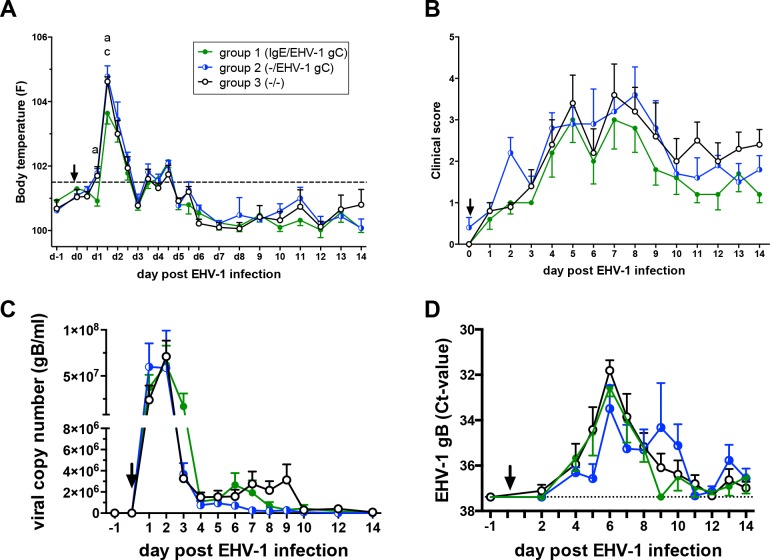
Body temperatures, clinical signs, nasal shedding and viremia after experimental infection of weanlings with the EHV-1 strain NY03. The groups (n = 5 per group) were established based on IgE-bio and/or EHV-1 gC antigen treatments of the foals at birth: group 1 received IgE-bio and Sav-gC/IL-4 antigen; group 2 received Sav-gC/IL-4 antigen; control group 3 no treatment at birth. EHV-1 challenge infection occurred at seven months of age (day 0; arrow) and was performed under identical conditions for all three groups. On day 0, measurements and samples were taken before infection. (A) Body temperatures; the dotted horizontal line shows the cut-off value for fever (101.5°F); (B) clinical scores; (C) nasal shedding expressed as viral copy numbers of the EHV-1 gene gB per ml nasal secretion sample and (D) viremia (gB Ct-value) per 5 x 10^6^ PBMC. Nasal shedding and viremia were evaluated by real-time PCR. The dotted horizontal line shows the positive PCR Ct-value cut-off value. All negative PCR values were set to this value. All graphs show means and standard errors per group. Significant differences between groups: a = groups 1 and 3; c = groups 1 and 2.

Experimental infection with EHV-1 strain NY03 at weanling age resulted in a short bi-phasic fever in all weanlings. Body temperatures were lower in weanling group 1 (IgE-bio and Sav-gC/IL-4 at birth) compared to the non-vaccinated control group 3 at 24 hours and 36 hours pi (both p<0.05). At the latter time point, the body temperature of group 1 weanlings was also lower than in group 2 (Sav-gC/IL-4 at birth) (p<0.01). This resulted in a lower initial fever peak in group 1 weanlings compared to the other two groups in response to EHV-1 infection (Fig 1A). All foals showed serous to mildly discolored nasal secretions, and slightly to moderately enlarged mandibular lymph nodes. Eye discharge and neurological signs were not observed. Total clinical scores did not differ between groups (Fig 1B).

Quantitative PCR of the EHV-1 gB gene was performed to determine EHV-1 DNA in nasal secretions and PBMC of the weanlings. EHV-1 DNA was not detected prior to EHV-1 infection. In the nasal secretion, EHV-1 DNA peaked on day 2 pi, declined to low positive values between days 3–9 pi, and were still not completely negative by d14 pi (Fig 1C). Viremia was detectable in all three groups, peaked on day 6 pi, became negative for most weanlings by d11pi but was still detectable in some horses on d14pi (Fig 1D). Significant differences in the quantity of EHV-1 DNA in nasal secretion or in PBMC were not observed between the groups.

### Antibodies to EHV-1 gC and gD in serum

**Fig 2:**
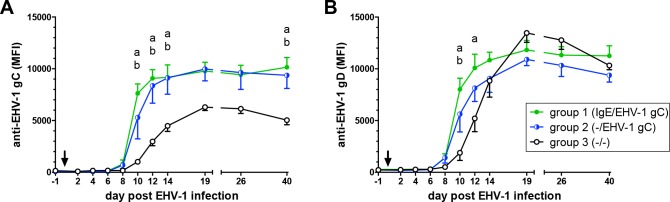
Antibodies to gC and gD in serum of weanlings after experimental EHV-1 infection (day 0; arrow). The weanling groups correspond to IgE-bio and/or EHV-1 gC antigen treatments during the neonatal period. At weanling age all three groups of weanlings were infected with the same dose of EHV-1 strain NY03. Antibodies in serum were determined by a EHV-1 Multiplex assay. The graphs show: (A) total serum anti-EHV-1 gC antibodies and (B) total serum anti-EHV-1 gD antibodies. The graphs represent means and standard errors by group. Significant differences between groups: a = groups 1 and 3; b = groups 2 and 3.

Anti-gC and anti-gD total Ig in serum did not differ between groups 1 and 2 at any time (Fig 2). In both vaccinated groups, anti-gC total Ig was significantly increased on days 10–14 pi compared to group 3 (p-values <0.01–0.0001) indicating an earlier onset and higher magnitude of EHV-1-specific antibodies in the foals receiving the neonatal EHV-1 gC/IL-4 treatment. The serum anti-gC total Ig of group 1 and 2 foals then reached a plateau. The serum antibodies in the group 3 weanlings began to rise on day 10 to 19 pi and afterwards reached a lower mean anti-gC antibody plateau than the two vaccinated groups. Group 3 anti-gC Ig values were similar to those in groups 1 and 2 on days 19 and 26 pi. However, group 3 anti-gC antibodies declined again to a significantly lower anti-gC Ig value than the two neonatal vaccination groups on day 40 pi (group 1: p<0.001, group 2: p<0.01) (Fig 2A). Although group 1 and 2 neonates were not vaccinated after birth against EHV-1 gD, anti-gD total Ig responses also increased earlier in the vaccinated foals than in non-vaccinated controls. This bystander effect was observed for group 1 on day 10 (p<0.001) and 12 pi (p<0.01), and for group 2 foals on day 10 pi (p<0.05). Thereafter, anti-gD total Ig values in serum were similar between all groups (Fig 2B). Total anti-gC antibodies were measured beyond day 40 pi in monthly intervals until day 220 pi. Antibody values slowly decreased over time in all groups and were still clearly detectable nine months post infection (data not shown). The earlier onset and quantitatively higher anti-gC antibody responses shortly after EHV-1 infection suggested that groups 1 and 2 had a pre-existing anti-gC memory B-cell population as a result of the neonatal EHV-1 gC/IL-4 vaccination.

### Serum isotype responses to EHV-1 gC

**Fig 3:**
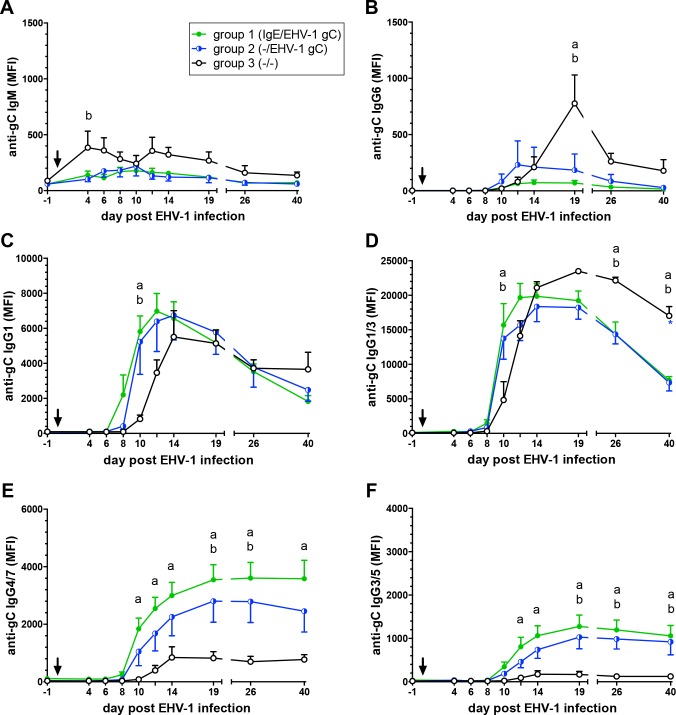
Anti-gC isotypes in the serum of weanlings after experimental EHV-1 infection (arrow). Weanlings in group 1 received IgE and EHV-1 gC antigen at birth. Weanlings in group 2 received EHV-1 gC antigen at birth. Weanlings in the control group 3 did not receive any treatment at birth. Antibody isotypes to gC were measured in a EHV-1 Multiplex assay. (A) anti-IgM; (B) anti-IgG6; (C) anti-IgG1; (D) anti-IgG1/3; (E) anti-IgG4/7; (F) anti-gC IgG3/5. The graphs show means and standard errors by group. Significant differences between groups: a = groups 1 and 3; b = groups 2 and 3.

If anti-gC memory B-cells existed in the neonatal vaccination groups 1 and 2 at the time of infection, an earlier onset and higher magnitude IgG isotype and reduced IgM response would have been expected compared to group 3. This was observed after EHV-1 challenge infection (Fig 3). Anti-gC IgM antibodies in the vaccinated groups did not increase beyond their baseline value (Fig 3A). Although the primary IgM response in group 3 was low, an initial anti-gC IgM increase was observed in the non-vaccinated group 3 on day 4 pi (p<0.05). The initial anti-gC antibody response in serum after EHV-1 challenge was mainly composed of IgG1 and IgG1/3 antibodies (Fig 3C and 3D). In the two vaccinated groups, anti-gC IgG1 and IgG3 values were higher than in group 3 on day 10 pi (IgG1: p<0.01; IgG1/3 p<0.001) supporting that these two isotypes contributed to the early onset of the antibody response in the vaccinated weanlings. After day 10 pi, anti-gC IgG1 values were similar in all groups, peaked on days 12–14 pi and then declined (Fig 3C). Anti-gC IgG1/3 serum antibody values mimicked the IgG1 response for weanlings in groups 1 and 2. However, they were overall higher due to the detection of both IgG1 and IgG3 isotypes by the antibody used. This suggested that anti-gC IgG3 was also produced in response to EHV-1 infection. Group 3 serum anti-gC IgG1/3 peaked on day 19 pi and was increased compared to the two vaccinated groups on days 26 (p<0.01) and 40 pi (p<0.001) suggesting an increased production of IgG3 in group 3 that was not noted in the other two groups (Fig 3D). Similarly, group 3 weanlings responded with low but significantly increased anti-gC IgG6 on day 19 pi (p<0.0001), while IgG6 was low in groups 1 and 2 throughout the study (Fig 3B).

Anti-gC IgG4/7 (Fig 3E) overall mimicked the total anti-gC Ig production (Fig 2A). IgG4/7 antibodies were detectable on day 10 pi in the vaccinated groups 1 and 2, increased until day 19 pi, and were then maintained at a plateau level. Group 3 anti-gC IgG4/7 was detectable on day 12 pi, increased slightly by day 14pi, and remained at this level for the rest of the study period. Compared to group 3, increased IgG4/7 values were observed between days 10–40 pi in group 1 (p-values <0.05–0.0001) and on days 19 and 40 pi in group 2 (p<0.05 and 0.01, respectively (Fig 3E). Serum anti-gC IgG3/5 antibodies were overall low and almost undetectable in group 3 (Fig 3F). In groups 1 and 2, the amount of anti-gC IgG3/5 was of lower compared to the other isotypes, showed a similar profile as IgG4/7 antibodies, and were significantly increased compared to group 3 on days 12–40 pi (p-values <0.05–0.0001). Serum IgA antibodies were almost undetectable in all groups (data not shown).

Overall, the serum anti-gC isotype values supported the earlier onset and overall increased antibody responses to EHV-1 challenge infection in weanlings that were vaccinated with gC/IL-4 after birth compared to non-vaccinated group 3 foals. Vaccinated groups were lacking an IgM response and developed a broad serum anti-gC IgG response with an IgG1/3/4/7/5^low^ serum isotype profile after challenge infection, while the non-vaccinated weanlings responded with an initial weak IgM increase followed by a narrow IgG isotype response with an IgG1/3/4^low^/6^low^/7^low^ profile and a delayed onset of all these IgG antibodies.

### Cytokine secretion from PBMC after EHV-1 re-stimulation

**Fig 4:**
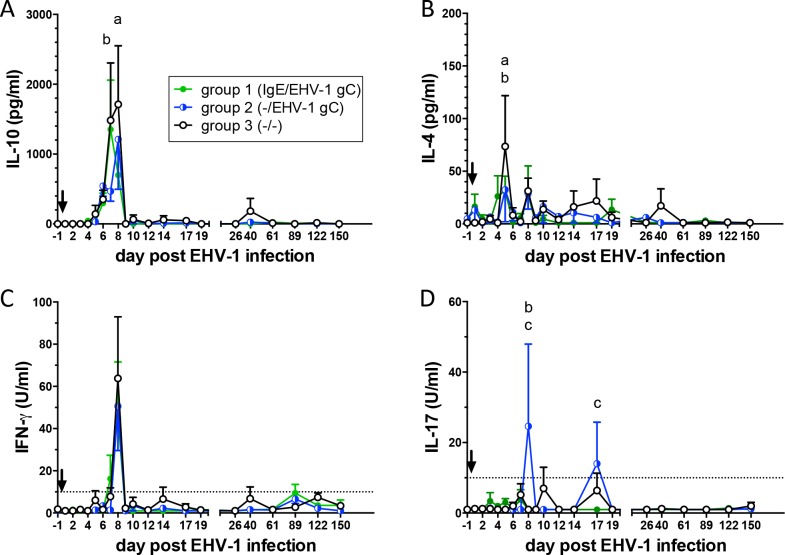
Cytokine secretion from PBMC of weanlings after experimental EHV-1 infection *in vivo* (arrow). Weanlings in group 1 received IgE and EHV-1 gC antigen as neonates and weanlings in group 2 received EHV-1 gC antigen. Weanlings in the control group 3 did not receive any neonatal. PBMC were isolated up to 5 months post EHV-1 infection. Cells were re-stimulated *ex vivo* with EHV-1 for 48 hour to provoke cytokine production. PBMC in medium served as non-stimulated control. Cytokines in cell culture supernatants were determined by cytokine multiplex analysis. The cytokine secretion values in graphs A-D were corrected by the non-stimulated control values of each weanling and day. The graphs show means and standard errors by group. Significant differences between groups: a = groups 1 and 3; b = groups 2 and 3; c = groups 1 and 2.

Cellular immune responses from EHV-1 infected weanlings were measured before and up to five months post EHV-1 infection. PBMC were re-stimulated with EHV-1 *ex-vivo*. Secreted IL-10, IL-4, IFN-γ, and IL-17 were evaluated in PBMC supernatants by multiplex cytokine analysis. EHV-1 re-stimulation induced substantial IL-10 and low IL-4 secretion from PBMC (Fig 4A and 4B). In contrast to the control group, PBMC from both neonatal vaccination groups showed significantly reduced IL-10 secretion on day 7 pi (group 2, p<0.05) or day 8 pi (group 1, p<0.05) and reduced IL-4 secretion on day 5 pi (both p<0.05). EHV-1 re-stimulation resulted in a sharp IFN-γ secretion peak on day 8 pi in all groups (Fig 4C). IL-17 secretion from PBMC was overall low (Fig 4D). However, IL-17 was increased on day 8 pi in group 2 compared to the other two groups (both, p<0.0001) and in group 2 compared to group 1 on d17 pi (p<0.05).

In general, EHV-1 re-stimulation of PBMC resulted in a peak of cytokine secretion within the first 5–8 days pi, which corresponded to peak viremia (day 6 pi). Thereafter, EHV-1 re-stimulation induced only very low levels of secreted cytokines. At all time points, PBMC aliquots were simultaneously stimulated with PMA and ionomycin which provoked high concentrations of all four cytokines from these cells at all time points (data not shown). The finding supports the high immune regulatory capacity of EHV-1 and its ability to effectively down-regulate EHV-1-specific responses in immune cells.

### Cytokines produced by T-cells

**Fig 5:**
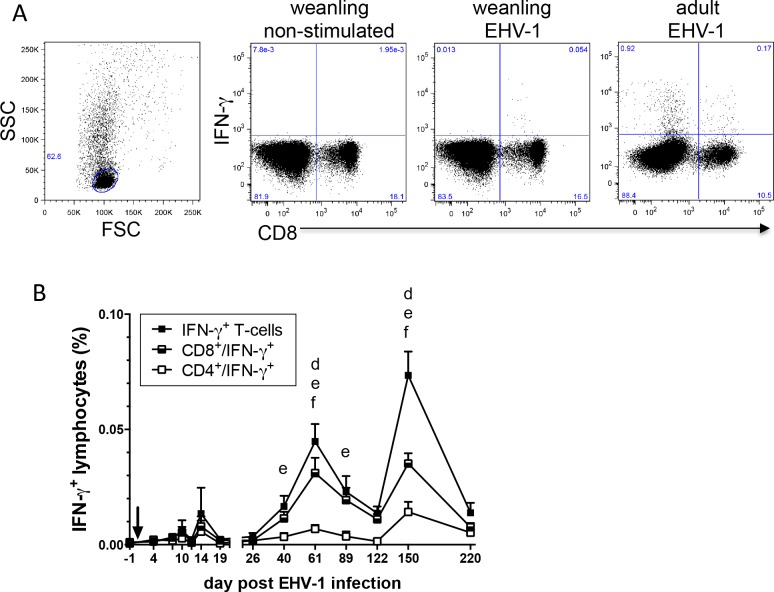
EHV-1-specific IFN-g producing T-cells in weanlings (n = 15) after EHV-1 infection (arrow). PBMC were isolated at different times after infection and were re-stimulated for 48hours with EHV-1 (MOI = 1). Cells were fixed, stained for intracellular IFN-g and cell surface CD4 and CD8 expression, and were analyzed by flow cytometry. A) Representative images of IFN-g producing CD8+ T-cells after EHV-1 stimulation. The images from left to right show the cell morphology and the lymphocyte gate; weanling PBMC on day 61 pi cultured in medium; PBMC from the same weanling re-stimulated with EHV-1; and EHV-1 re-stimulated cells from an adult horse EHV vaccinated horse. B) Total EHV-1-specic IFN-g producing T-cells and CD4+ or CD8+ IFN-g producing T-cells in the peripheral blood of the 15 weanlings until day 220 pi. Significant increases compared to the pre-infection control: d = total IFN-g T-cells, e = CD8+/IFN-g+, f = CD4+/IFN-g+.

EHV-1-specific, CD4^+^ and CD8^+^ T-cells were stained for intracellular cytokines and analyzed by flow cytometry. IL-4, IL-10 or IL-17 were not detectable in T-cells (data not shown). IFN-γ producing T-cells were observed after EHV-1 infection without significant differences between groups (Fig 5A). Less than 0.1% EHV-1-specific IFN-γ producing T-cells were detected at all time points until 9 months pi (Fig 5B). This was in sharp contrast to IFN-γ production from EHV-1-specific T-cells in adult horses previously exposed to EHV-1 as shown in Fig 5A and reported earlier [40]. Nevertheless, total IFN-γ producing EHV-1-specific T-cells appeared in the peripheral blood in spikes with increasing percentages at days 14, 61 and 150 pi. Total IFN-γ producing T-cells were elevated compared to the pre-infection percentages on days 61 pi (p<0.001) and 150 pi (p<0.0001). The majority of the EHV-1-specific IFN-γ producing T-cells were CD8^+^ cells. Consequently, CD8^+^/IFN-γ^+^ cells also peaked on day 61 and 150 pi (both p<0.0001) and were increased compared to pre-infection values on days 40 and 89 pi (both p<0.05). Despite their overall very low percentages, CD4^+^/IFN-γ^+^ T-cells were increased on days 61 (p<0.05) and 150 pi (p<0.01) compared to the pre-infection time point.

Overall, the appearance of EHV-1-specific T-cell immunity was late (day 40 pi) compared to EHV-1 antibody induction that was detectable on day 8–10 pi (Figs 2 & 3). In addition, the lack of detectable IL-4, IL-10, IL-17 and IFN-γ producing T-cells in the circulating PBMC until day 8 pi further suggested that the observed secretion of these cytokines from PBMC between days 5–8 pi (Fig 4) originated from innate immune cells rather than EHV-1-specific T-cells.

## Discussion

Neonatal B-cell functions and antibody responses to traditional vaccines are reduced compared to adults [14,42,43]. Neonatal vaccination is desirable against many pathogens, including EHV-1. It is, however, still a challenge due to the intrinsic functions of the neonatal immune system such as anti-inflammatory rather than pro-inflammatory responses to innate immune stimuli, and a trend to immune regulation rather than activation of effector functions and adaptive immune memory [43]. Coming from the low antigenic environment of the uterus, the neonatal immune system has to adapt rapidly to multiple environmental and antigenic challenges after birth. It is thus not surprising that immune regulation and tolerance to harmless commensal bacteria or environmental and food antigens dominate the immune response early in life. In humans and rodent models, neonatal immune responses are characterized by limited plasma cell and germinal center B-cell responses [44]. Neonatal foals also respond poorly with low antibody amounts to vaccination. Nevertheless, foals start to produce several IgG isotypes almost immediately after birth [14].

The goal of this neonatal vaccination approach against EHV-1 was to find evidence for the induction of adaptive B-cell immunity by stimulating innate IL-4 production in basophils and to use this pathway as a potential new EHV-1 vaccination strategy for neonatal foals. We performed a first vaccination trial using three groups of neonates receiving either (1) IgE-bio and Sav-gC/IL-4 to stimulate IL-4 production from basophils and activate gC-specific B-cells, or (2) Sav-gC/IL-4 only to directly activate B-cells without the basophil IL-4 stimulus, or (3) no neonatal treatment in the control group. We then tested the ability of the neonatal vaccine to protect these foals from EHV-1 infection and evaluated their humoral and cellular immune responses.

Clearly, the neonatal EHV-1 gC vaccine used here did not induce any systemically detectable gC-specific antibodies after neonatal vaccination which would have been a prerequisite for a protective EHV-1 vaccine as previously shown in older horses [45,46]. It can be concluded that the induction of antibodies directly after birth would likely require additional components or an adjuvant to be added to the vaccine formula. These components would also need to target innate immune pathways that neonates can perform. Foals almost immediately start IgG production after birth [14]. In particular, they make considerable amounts of endogenous IgG1 and IgG3 antibodies early in life [15,16]. The exact mechanisms by which B-cells of very young foals are activated to produce IgG in the absence of Th2-immunity are currently unknown and still need to be unraveled.

In agreement with the missing antibody response after neonatal vaccination was the finding that none of the weanling groups were fully protected from EHV-1 infection after challenge. All weanlings developed a fever, mild respiratory clinical signs, nasal EHV-1 shedding and viremia. All of these parameters observed in weanlings from EHV-1 naïve mares were similar to those described in previous EHV-1 infection studies in older horses [45–47]. Neurological signs were not expected after infection with the non-neurogenic NY03 strain and were not observed. It can be concluded that the missing maternal EHV-1 specific antibody transfer at birth did not modify clinical signs or severity of the disease in these weanlings compared to previously performed EHV-1 infection studies in older horses or horses that likely received maternal EHV-1 specific antibodies.

Despite the lack of antibody production immediately after neonatal vaccination, the experimental infection at weanling age with EHV-1 revealed differences in the foals receiving the neonatal EHV-1 gC vaccine compared to the non-vaccinated controls. First, weanlings receiving IgE-bio and Sav-gC/IL-4 had a significantly lower fever peak after infection indicating the induction of partial protection from EHV-1 infection in this vaccinated group.

In addition and most remarkably, the two vaccinated weanling groups developed an earlier and quantitatively higher anti-gC antibody response after EHV-1 challenge compared to non-vaccinated weanlings. Antibody responses in the vaccinated groups were independent of the oral IgE-bio treatment at birth. This suggested that a single Sav-gC/IL-4 injection on day 2 of life is able to induce neonatal B-cell activation. We hypothesize that neonatal B-cell activation happens for an EHV-1 gC-specific B-cell by crosslinking of gC-specific B-cell receptors and simultaneous binding of the IL-4 portion of the Sav-gC/IL-4 fusion protein to the IL-4-receptor on the same B-cell. Our results support that this T-cell independent activation mechanism of equine neonatal gC-specific B-cells likely resulted in a pre-existing memory B-cell population that was still present at seven months of age when the EHV-1 infection occurred.

After EHV-1 challenge, the vaccinated groups responded immediately with a serum IgG response composed of anti-gC IgG1/3/4/7/5^low^ isotypes. The missing IgM response in the vaccinated groups further supported the induction of gC-specific IgG memory B-cells by the Sav-gC/IL-4 antigen at birth. In contrast, non-vaccinated weanlings showed a classical initial antibody response to EHV-1, characterized by short-lasting IgM antibodies and followed by a longer-lasting IgG response against EHV-1 gC. The IgG antibody response of non-vaccinated weanlings was dominated by IgG1 and IgG3, low IgG4/7 and IgG6, with little to no IgG5 and was of overall lower isotype variety in comparison to the vaccinated groups. In horses, primary IgM response are often low (unpublished observations) and some of the classical IgM functions of other species seem to be compensated by equine IgG1 which is the first IgG isotype observed after infection with many equine pathogens [14] including the EHV-1 infection performed here.

The induction of gC-specific memory B-cells by the neonatal vaccine and in particular by the single dose injection of Sav-gC/IL-4 antigen still requires confirmation and direct poof of the existence of these memory B-cells in foals. This is currently a difficult task due to the lack of memory B-cell specific markers in horses. We have evaluated several memory B-cell markers directed against mouse and human CD molecules without much success (data not shown). The identification of memory B-cells in horses thus has to be delayed until a specific marker for these cells becomes available. However, data from mice indicated that most activated neonatal B-cells differentiated in extrafollicular pathways outside of the germinal centers of lymphatic tissues. The extrafollicular activation resulted in a quietly residing memory B-cell population but did not induce IgG producing long-lived plasma cells [43]. A similar mechanism may have occurred here after vaccination with Sav-gC/IL-4 antigen on day 2 of life. This would offer an explanation for the earlier and increased antibody responses in vaccinated foals after EHV-1 challenge despite the missing IgG production directly after vaccination.

It was not expected that the neonatal vaccine would affect T-cell responses or other cytokine responses other than IL-4. After EHV-1 challenge, the evaluation of cellular immune parameters in re-stimulated PBMC confirmed that cytokine and T-cell responses showed no or minor differences in the vaccinated and non-vaccinated foals. However, the longitudinal analysis of EHV-1-specific cellular immunity after infection showed several novel results that have not been reported previously. Most strikingly was the sharp increase of cytokine responses, including IL-10 and IFN-γ secretion, from EHV-1 re-stimulated PBMC by the end of the first week pi. These IL-10 and IFN-γ peaks were followed by a rapid down-regulation of cytokine secretion and a non-responsiveness of PBMC to EHV-1 re-stimulation by and after day 9 pi. In addition, EHV-1-specific T-cell responses in the EHV-1 infected weanlings were low and almost undetectable until d40 pi. Afterwards, T-cell responses peaked and declined over time and also shifted from the initial CD8^+^ phenotype on day 61 pi to a CD4^+^ and CD8^+^ phenotype by day 150 pi. These findings confirm the high immune regulatory potential of EHV-1, its ability to induce systemic suppression of cytokine production and to effectively down-regulate and modulate T-cell responses.

## Conclusions

The novel neonatal vaccination approach described here induced improved antibody responses and partial protection after foals were challenged by EHV-1 infection. It can be concluded that the neonatal vaccine, through basophil-derived IL-4 and/or direct gC/IL-4 antigen-mediated activation of neonatal B-cells, induced a memory B-cell response early in life. Although detectable antibody production was missing after vaccination, it is still notable that a single gC/IL-4 antigen injection without adjuvant is sufficient to induce a memory B-cell response in neonatal foals. These gC-specific memory B-cells were still functional by the time of EHV-1 challenge at 7 months of age. For the development of a fully protective neonatal EHV-1 vaccine additional vaccine components need to be added to further support the existing neonatal immune functions and to induce EHV-1 antibody production early in life.

## Supporting Information

S1 Fig Conjugation confirmation and functional testing of the neonatal vaccine components biotinylated IgE (IgE-bio) and streptavidin-conjugated gC/IL-4 (Sav-gC/IL-4). (A) Percentages of IgE^+^ cells in the MHCII^low^ fractions of 8 adult mares. MHCII^low^ cell fractions were obtained by MHCII depletion sorting. Afterwards, surface IgE^+^ cells were stained using one aliquot of the MHCII^low^ cells and flow cytometric analysis. (B) Remaining MHCII^low^ cells were incubated for 20 hours in medium or with increasing concentrations of IgE-bio. Cells were harvested, stained for cell surface-bound IgE-bio using Sav-Cy5, and were analyzed by flow cytometry. (C) Sav-gC/IL-4 was coated to an ELISA plate in different concentrations and detected with biotin-conjugated peroxidase. After substrate addition, colorimetric development was measured. (D) MHCII^low^ cells from 4 mares were incubated for 20 hours in medium, or with 1 or 3 ug/ml IgE-bio. Afterwards, all cell culture supernatants were replaced by medium containing Sav-peroxidase. Cells were incubated for another 24 hours. Then, supernatants were harvested and IL-4 secretion was quantified in a bead-based assay. Horizontal bars in the graphs show medians. Significant increases in IgE-bio binding in (B) and IL-4 secretion in (D) compared to the medium controls: * p<0.05; *** p<0.001.
(PDF)

S2 Fig IL-4 secretion from basophils in neonatal foals. Neonatal foals from EHV-1 naïve mares were divided in three treatment groups (n = 5). Foals in group 1 received IgE-bio at birth and Sav-gC/IL-4 antigen on day 2 of age. Foals in group 2 received Sav-gC/IL-4 on day 2. Foals in control group 3 did not receive any treatment after birth. MHCII^low^ cells were obtained by depletion sorting of neonatal foal PBMC on days 2 and 5 of life. Blood samples on day 2 were obtained before Sav-gC/IL-4 was administrated. MHCII^low^ cells were either kept in medium, or stimulated with Sav-gC/IL-4 or anti-IgE in the presence of the secretion blocker Brefeldin A for 4 hours. Cells were stained afterwards for intracellular IL-4 and cell surface IgE and measured by flow cytometric analysis.
(PDF)

S1 File Supporting materials and methods. (DOCX)

S2 File Supporting results. (DOCX)

## Abbreviations

gC: glycoprotein C
gC/IL-4: gC/IL-4 fusion protein
gD: glycoprotein D
EHV-1: equine herpesvirus type 1
EqE 37–1: equi-murine heterohybridoma cell line expressing equine IgE
IFN-γ: interferon-gamma
Ig: immunoglobulin
IgE: immunoglobulin E
IgE-bio: biotinylated IgE
IgG: immunoglobulin G
IL-4: interleukin-4
IM: intramuscular
MFI: median fluorescence intensity
MOI: multiplicity of infection
PBMC: peripheral blood mononuclear cells
PBS: phosphate buffered saline
pi: post infection
Sav: streptavidin
Sav-gC/IL-4: streptavidin-conjugated gC/IL-4 fusion protein
USDA APHIS: United States Department of Agriculture: Animal and Plant Health Inspection System
Th cell: T-helper cell
