# Supplementary figures and images for: Neonatal Immunization with a Single IL-4/Antigen Dose Induces Increased Antibody Responses after Challenge Infection with Equine Herpesvirus Type 1 (EHV-1) at Weanling Age
Source: PLoS One. 2017 Jan 3;12(1):e0169072. doi: 10.1371/journal.pone.0169072 (PMC5207648; doi:10.1371/journal.pone.0169072)

**S1 Figure:** Conjugation and functional testing of the neonatal vaccine components.

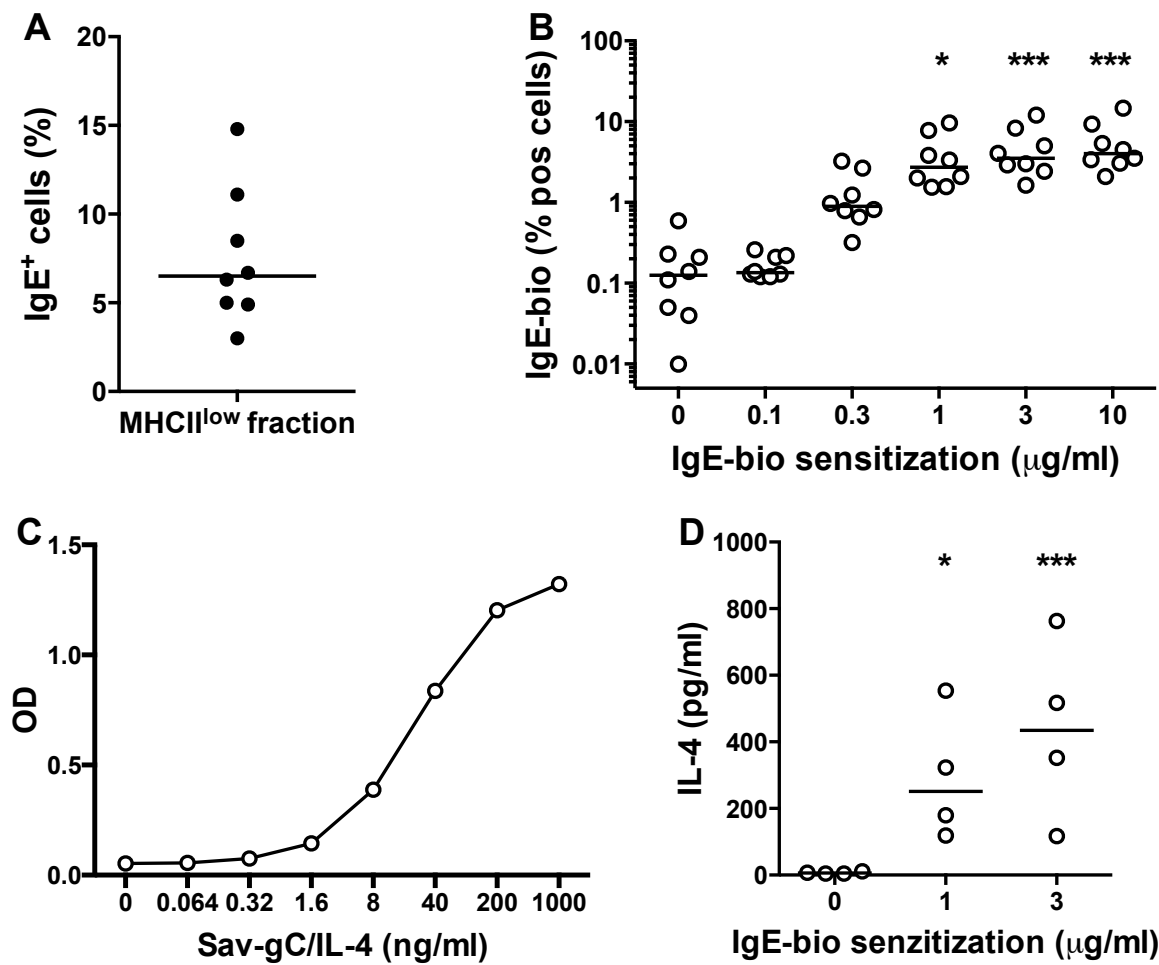

Supplement: S1 Fig — (A) Percentages of IgE+ cells in the MHCIIlow fractions of 8 adult mares. MHCIIlow cell fractions were obtained by MHCII depletion sorting. Afterwards, surface IgE+ cells were stained using one aliquot of the MHCIIlow cells and flow cytometric analysis. (B) Remaining MHCIIlow cells were incubated for 20 hours in medium or with increasing concentrations of IgE-bio. Cells were harvested, stained for cell surface-bound IgE-bio using Sav-Cy5, and were analyzed by flow cytometry. (C) Sav-gC/IL-4 was coated to an ELISA plate in different concentrations and detected with biotin-conjugated peroxidase. After substrate addition, colorimetric development was measured. (D) MHCIIlow cells from 4 mares were incubated for 20 hours in medium, or with 1 or 3 ug/ml IgE-bio. Afterwards, all cell culture supernatants were replaced by medium containing Sav-peroxidase. Cells were incubated for another 24 hours. Then, supernatants were harvested and IL-4 secretion was quantified in a bead-based assay. Horizontal bars in the graphs show medians. Significant increases in IgE-bio binding in (B) and IL-4 secretion in (D) compared to the medium controls: * p<0.05; *** p<0.001. (PDF) [file pone.0169072.s001.pdf]

**S2 Figure:** IL-4 secretion from basophils in neonatal foals.

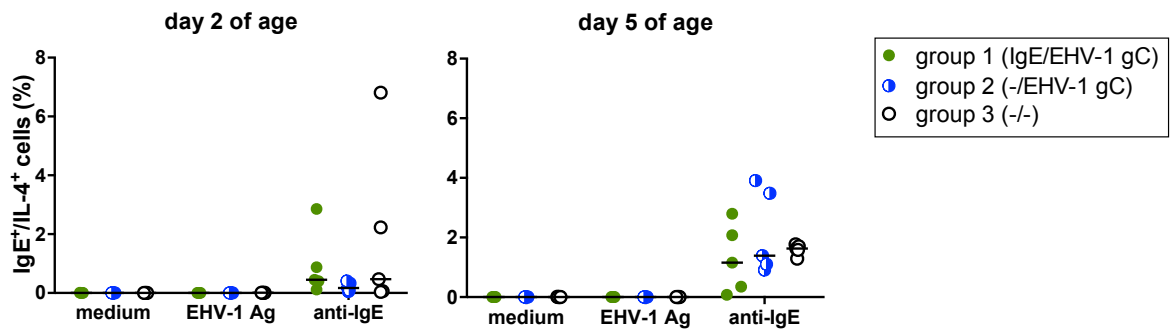

Supplement: S2 Fig — Neonatal foals from EHV-1 naïve mares were divided in three treatment groups (n = 5). Foals in group 1 received IgE-bio at birth and Sav-gC/IL-4 antigen on day 2 of age. Foals in group 2 received Sav-gC/IL-4 on day 2. Foals in control group 3 did not receive any treatment after birth. MHCIIlow cells were obtained by depletion sorting of neonatal foal PBMC on days 2 and 5 of life. Blood samples on day 2 were obtained before Sav-gC/IL-4 was administrated. MHCIIlow cells were either kept in medium, or stimulated with Sav-gC/IL-4 or anti-IgE in the presence of the secretion blocker Brefeldin A for 4 hours. Cells were stained afterwards for intracellular IL-4 and cell surface IgE and measured by flow cytometric analysis. (PDF) [file pone.0169072.s002.pdf]
