# Supplementary material for: Neonatal Immunization with a Single IL-4/Antigen Dose Induces Increased Antibody Responses after Challenge Infection with Equine Herpesvirus Type 1 (EHV-1) at Weanling Age
Source: PLoS One. 2017 Jan 3;12(1):e0169072. doi: 10.1371/journal.pone.0169072 (PMC5207648; doi:10.1371/journal.pone.0169072)
Supplement: S1 File — (DOCX) [file pone.0169072.s003.docx]

**S1 File**

**Supporting materials and methods**

**Biotinylation of IgE (IgE bio)**

Equine IgE originated from the equi-murine heterohybridoma cell line EqE 37-1 described previously [32]. Equine IgE was purified from serum-free EqE 37-1 cell culture supernatant using an anti-IgE affinity column [33] and a FPLC instrument (GE Heathcare, Piscataway, NJ). The protein concentration of purified equine IgE was determined in a BCA assay (Pierce, Rockford, IL). Purified IgE was then biotinylated using Sulfo-NHS-Biotin (Pierce, Rockford, IL) according to the manufacturer's instructions.

**Streptavidin-conjugated recombinant EHV-1 glycoprotein C (Sav-gC/IL-4)**

EHV-1 gC antigen was expressed in mammalian cells as an IL-4 fusion protein as previously described [26]. Briefly, the extracellular regions of gC (corresponding to amino acid residues 30-431 of EHV-1 strain Ab4; NCBI accession AY665713) were cloned into a mammalian pcDNA3.1-based vector (Invitrogen, Carlsbad, CA) containing the equine IL-4 gene as a tag for detection and purification as previously described [34]. Chinese hamster ovary cells were transfected with purified linear DNA from the expression construct. A stable, gC/IL-4 secreting cell line was obtained after neomycin selection and limiting dilution cloning [25]. Then, gC/IL-4 was purified from serum-free supernatant using a FPLC instrument (GE Healthcare, Piscataway, NJ) and an anti-IL-4 affinity column [34]. Purified gC/IL-4 antigen was conjugated with Sav-hydrazide (Pierce, Rockford, IL) following the manufacturer’s instructions.

The Sav conjugation was confirmed by ELISA. Nunc brand polystyrene plates (Thermo Scientific, Rochester, NY) were coated with Sav-conjugated gC/IL-4 (Sav-gC/IL-4) in concentrations between 64 pg/ml and 1 µg/ml in carbonate buffer (1M NaHCO_3_, 1M Na_2_CO_3_, pH 9.6) and were incubated overnight at 4°C. Plates were washed four times with phosphate buffered saline, 0.05% Tween20 (Sigma-Aldrich, St. Louis, MO), followed by incubation with biotin-peroxidase (Pierce, Rockford, IL) at a dilution of 1:10,000 for 30 minutes. After another four washes, substrate buffer (33.3 mmol citric acid, 66.7mmol NaH_2_PO_4_, pH 5.0), combined with 130μg/mL 3,3’,5,5’ Tetramethylbenzidine (Sigma-Aldrich, St. Louis, MO) and 0.012% hydrogen peroxide was added to the plate. The reaction was stopped after 15 minutes using 1N H_2_SO_4_. Plates were measured using an automatic plate reader (Biotek, Winooski, VT) at 450 nm absorbance.

**Isolation of PBMC and MHC class II^low^ (MHCII^low^) cells**

PBMC were isolated from heparinized blood samples by density gradient centrifugation (Ficoll-Paque^TM^ Plus, GE Healthcare, Piscataway, NJ). Equine MHCII^low^ cells, containing an enriched fraction of surface IgE^+^ basophils, were obtained by a MHCII depletion sorting using magnetic beads as previously described in detail [17]. To determine the original percentages of IgE^+^ cells in the MHCII^low^ fractions, one aliquot of the MHCII^low^ cells from each horse was stained immediately after sorting with Alexa 647 conjugated anti-IgE 176 diluted 1:100 in cold PBS/BSA (PBS with 0.5% (w/v) BSA and 0.02% (w/v) sodium azide, 4°C) for 10 min on ice. Cells were washed once with cold PBS/BSA and analyzed by flow cytometry in a FACS Canto II flow cytometer (BD Biosciences, San Diego, CA).

**Confirmation of IgE biotinylation, IgE receptor binding and IL-4 secretion induced by Sav crosslinking *in vitro***

The ability of IgE-bio to bind to cellular IgE receptors was tested using MHCII^low^ cells from eight adult Icelandic mares. MHCII^low^ cells were re-suspended in cell culture medium (DMEM containing 10% (v/v) fetal calf serum, 1% (v/v) non-essential amino acids, 2 mM L-glutamine, 50 µM 2-mercaptoethanol, 50 µg/ml gentamycin; all from Gibco, Invitrogen, Grand Island, NY). A total of 6x10^5^ MHCII^low^ cells per well were seeded into sterile 96 well plates and incubated with IgE-bio ranging from 0.1 to 10 µg/ml at 37°C in a humidified 5% CO_2_ incubator. Aliquots of cells in medium served as controls. After 20 hours, cells were harvested, washed once with cold PBS/BSA, and stained with Sav-Cy5 (Amersham Biosciences, Little Chalfont, UK) for 10 minutes on ice. Cells were washed with cold PBS/BSA one more time and were analyzed by flow cytometry.

Induction of IL-4 secretion by IgE-bio and Sav crosslinking was tested using MHCII^low^ cells from four adult Icelandic mares. MHCII^low^ cells were incubated with either 1 or 3 µg/ml IgE-bio or kept in medium for 20 hours of incubation as described above. Afterwards, the cell culture supernatant supernatants were replaced by medium containing Sav-peroxidase (0.5 µg/ml) and the cells were incubated for another 24 hours. Then, cell culture supernatants were harvested and IL-4 concentrations were measured using an IL-4 bead-based assay [38].

**Neonatal basophil stimulation assay**

Heparinized blood samples were obtained from neonatal foal on days 2 and 5 of life. Blood samples on day 2 were obtained before Sav-gC/IL-4 injection. PBMC isolation and MHCII^low^ cell depletion sorting were performed as described above. A total of 6x10^5^ cells MHCII^low^ cells were either kept in in cell culture medium alone, or were stimulated in medium supplemented 5 ug/ml Sav-gC/IL-4, or with 1ug/ml anti-IgE 134 in the presence of the secretion blocker Brefeldin A (10 μg/ml; Sigma, St. Louis, MO). After incubation for 4 hours at 37°C in a 5% CO_2_ incubator, the cells were washed in PBS and fixed in 2% formaldehyde for 20 minutes at room temperature. Cells were double stained for intracellular IL-4 using Alexa 647 conjugated anti-IL-4 clone 13G7 and for cell surface IgE using Alexa 488 conjugated anti-IgE 176 as previously described in detail [17]. The cells were measured by flow cytometry to analyze IgE^+^/IL-4^+^ cells before and after Sav-gC/IL-4 administration.

**Statistical analysis of post vaccination data**

D’Agostino & Pearson normality tests indicated that values on most days were not normally distributed. The binding of IgE-bio to equine MHCII^low^ cells and the IL-4 secretion after sensitization with IgE-bio and Sav stimulation was analyzed by non-parametric Friedman tests with Dunn’s multiple comparisons and by comparing each of the IgE-bio concentrations to the medium control. Serum antibody values before and after neonatal vaccination were expressed as fold-changes compared to the day 2 pre-vaccination serum value of each neonatal foal. Differences in antibodies values and percentages of IgE^+^/IL-4^+^ cells after *ex vivo* stimulation of neonatal MHCII^low^ cells between the three groups were analyzed by repeated-measures ANOVA with Bonferroni’s multiple comparison tests comparing neonatal vaccine groups 1 and 2 to the non-vaccinated group 3.
