# Supplementary material for: Neonatal Immunization with a Single IL-4/Antigen Dose Induces Increased Antibody Responses after Challenge Infection with Equine Herpesvirus Type 1 (EHV-1) at Weanling Age
Source: PLoS One. 2017 Jan 3;12(1):e0169072. doi: 10.1371/journal.pone.0169072 (PMC5207648; doi:10.1371/journal.pone.0169072)
Supplement: S2 File — (DOCX) [file pone.0169072.s004.docx]

**S2 File**

**Supporting results**

**Binding of IgE-bio to equine cells and IL-4 induction after IgE-bio crosslinking**

Binding of IgE-bio to cellular IgE receptors was tested by incubating the MHCII^low^ cell fraction of PBMC from eight adult mares with different concentrations of IgE-bio. Equine PBMC contain about 0.5-1% basophils with an IgE^+^/MHCII^low^ phenotype [17]. Directly after sorting, the MHCII^low^ cell fractions was enriched to 3-15% (median 6.5%; Suppl. Fig. 1A). After overnight incubation of the cells with IgE-bio, a dose-dependent binding was observed which was significantly increased compared to the medium control at 1 µg/ml or higher IgE-bio concentrations and reached a plateau between 3-10 µg/ml IgE-bio (Suppl. Fig. 1B). Different concentrations of Sav-gC/IL-4 were first tested by ELISA confirming the successful coupling of Sav to the EHV-1 gC antigen (Suppl. Fig. 1C). Afterwards, MHCII^low^ cells from four mares were sensitized with 1 and 3 µg/ml IgE-bio and then stimulated with Sav-peroxidase. Stimulation of IgE-bio sensitized cells induced a significant increase in IL-4 producing IgE^+^ cells compared to non-sensitized cells (Suppl. Fig. 1D).

**Influence of neonatal vaccination on EHV-1-specific serum antibodies and IL-4 production by basophils**

EHV-1 gC and gD-specific antibody values were measured in foal sera immediately after birth, before (day 2) and shortly after neonatal EHV-1 vaccination until foals were 3 months of age. Differences in gC or gD-specific antibodies were not observed between the vaccinated groups or the vaccinated and control groups at any of these time points (data not shown). Before IgE-bio administration and colostrum intake EHV-1 gC antibodies values of all foals ranged between 47-150 MFI (median 79 MFI) and gD antibodies between 58-160 MFI (median 71 MFI). Afterwards, group 1 foals received IgE-bio orally and all foals suckled colostrum ad lib. On day 2 after birth, gC (514-7176 MFI; median 2587 MFI), and gD antibodies (425-7881 MFI; median 1853 MFI) had increased in the serum of all foals. The increase in the antibody values after colostrum intake from EHV-1 naive mares was interpreted as non-specific due to the high increase in colostrum-derived serum antibody concentrations in the circulation of the neonates. The increase in serum gC- and gD-specific antibodies on day 2 further suggests some cross-reactive signals by EHV-4-specific antibodies from the colostrum. After sampling on day 2, group 1 and group 2 foals received EHV-1 antigen (Sav-gC/IL-4) intramuscularly. However, gC and gD serum antibody values had already decreased on day 5 after birth and continued to decline afterwards in all three groups.

The MHCII^low^ cell fraction was isolated by MHCII depletion sorting from foal PBMC on days 2 and 5 of age. The MHCII^low^ fraction contains equine neonatal basophils which can secrete IL-4 after stimulation via receptor bound IgE [17]. MHCII^low^ cells were stimulated with Sav-gC/IL-4 antigen *ex vivo* to test if the oral IgE-bio treatment and/or Sav-gC/IL-4 immunization resulted in detectable amounts of IL-4 secreting IgE^+^ cells. *In vitro* crosslinking of MHCII^low^ cells with anti-IgE induced comparable IL-4 production in neonatal basophils of all foal groups on days 2 and 5 of age. In contrast, Sav-gC/IL-4 stimulation did not result in detectable percentages of IgE^+^/IL-4^+^ cells (Suppl. Fig. 2). Similarly, IL-4 producing cells were undetectable in the non-stimulated MHCII^low^ cells of all groups.

Overall, the results showed that the expected IL-4 induction from neonatal basophils could be achieved by IgE-bio cross-linking *in vitro*. However, a direct *in vivo* effect of the IgE-bio and/or EHV-1 antigen administration on EHV-1-specific antibody production in foal groups 1 and 2 was not observed. The results further suggested that treating neonates with 1 mg IgE-bio orally and/or 0.5 mg Sav-gC/IL-4 IM is not sufficient to induce detectable amounts of IL-4 in circulating peripheral blood basophils or after *in vitro* Sav-gC/IL-4 stimulation. However, the results do not exclude that neonatal treatment with IgE-bio and/or Sav-gC/IL-4 antigen may have induced local effects at the site of antigen injection or in regional lymph nodes. To test the latter, an experimental challenge infection with EHV-1 was performed at weanling age.
